# Effects of aerobic exercise training on cerebral pulsatile hemodynamics in middle-aged adults with elevated blood pressure/stage 1 hypertension

**DOI:** 10.1152/japplphysiol.00689.2023

**Published:** 2024-04-11

**Authors:** Krista S. Reed, Abby M. Frescoln, Quinn Keleher, Angelique G. Brellenthin, Marian L. Kohut, Wesley K. Lefferts

**Affiliations:** Department of Kinesiology, Iowa State University, Ames, Iowa, United States

**Keywords:** arterial stiffness, cerebral pulsatility, exercise adaptations, hypertension, large artery hemodynamics

## Abstract

Mechanisms behind the protective effects of aerobic exercise on brain health remain elusive but may be vascular in origin and relate to cerebral pulsatility. This pilot study investigated the effects of 12-wk aerobic exercise training on cerebral pulsatility and its vascular contributors (large artery stiffness, characteristic impedance) in at-risk middle-aged adults. Twenty-eight inactive middle-aged adults with elevated blood pressure or stage 1 hypertension were assigned to either moderate/vigorous aerobic exercise training (AET) for 3 days/wk or no-exercise control (CON) group. Middle cerebral artery (MCA) pulsatility index (PI), large artery (i.e., aorta, carotid) stiffness, and characteristic impedance were assessed via Doppler and tonometry at baseline, 6, and 12 wk, whereas cardiorespiratory fitness (V̇o_2peak_) was assessed via incremental exercise test and cognitive function via computerized battery at baseline and 12 wk. V̇o_2peak_ increased 6% in AET and decreased 4% in CON (*P* < 0.05). Proximal aortic compliance increased (*P* = 0.04, partial η^2^ = 0.14) and aortic characteristic impedance decreased (*P* = 0.02, partial η^2^ = 0.17) with AET but not CON. Cerebral pulsatility showed a medium-to-large effect size increase with AET, although not statistically significant (*P* = 0.07, partial η^2^ = 0.11) compared with CON. Working memory reaction time improved with AET but not CON (*P* = 0.02, partial η^2^ = 0.20). Our data suggest 12-wk AET elicited improvements in central vascular hemodynamics (e.g., proximal aortic compliance and characteristic impedance) along with apparent, paradoxical increases in cerebral pulsatile hemodynamics.

**NEW & NOTEWORTHY** We identify differential central versus cerebrovascular responses to 12 wk of aerobic exercise training in middle-aged adults. Although proximal aortic compliance and characteristic impedance improved after 12 wk of exercise, cerebral pulsatility tended to unexpectedly increase. These data suggest short-term aerobic exercise training may lead to more immediate benefits in the central vasculature, whereas longer duration exercise training may be required for beneficial changes in pulsatility within the cerebrovasculature.

## Graphical abstract

**Figure:**
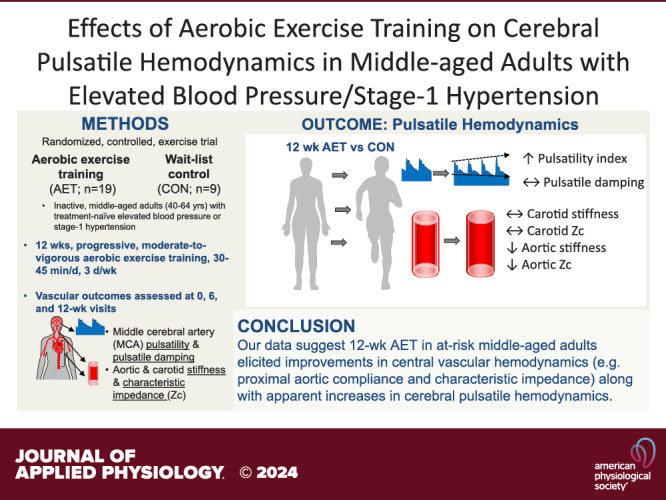

## INTRODUCTION

Prevalence of cerebrovascular and cognitive disease is anticipated to double every 20 years for the foreseeable future (1, 2). Prevention of these diseases is crucial in an aging population since there is currently no effective treatment (3, 4). Exercise has emerged as a potent and recommended lifestyle behavior to maintain brain health and prevent cerebrovascular and cognitive disease (4). Specifically, regular aerobic exercise has been shown to protect against cognitive decline (5–7), brain atrophy and damage (8–10), and cerebrovascular disease (11, 12). Although the exact mechanisms underlying the protective effects of aerobic exercise on the brain are currently unknown, they may be vascular in origin and relate to pulsatile blood flow patterns in the brain.

Cerebral pulsatility describes the discontinuous nature of blood flow in the brain that increases with age (13, 14) owing to *1*) increases in arterial stiffness and characteristic impedance that widen pulse pressure (15–17) and *2*) reductions in cerebrovascular pulsatile damping (18, 19). Greater pulsatile cerebral blood flow is associated with vascular and structural damage to the brain that ultimately contributes to cognitive dysfunction and cerebrovascular disease (17, 20, 21). Thus, cerebral pulsatility and its vascular contributors have emerged as key mechanisms of brain health and potential mechanism through which exercise protects the brain. Indeed, current data suggest higher cardiorespiratory fitness, attained through regular aerobic exercise, is beneficially associated with lower cerebral pulsatility (22, 23), arterial stiffness (24, 25), pulse pressure (26), and greater pulsatile damping (22). Recent data from randomized controlled exercise trials show 1 year of aerobic exercise training beneficially influences vascular contributors to cerebral pulsatility such as carotid stiffness and cerebrovascular impedance in cognitively normal (24, 26) and cognitively impaired (25) older adults. Interestingly, aerobic exercise training has only been shown to decrease cerebral pulsatility in cognitively impaired adults (25), with no effects seen in healthy older adults (24). These recent findings regarding aerobic exercise training and cerebral pulsatility are limited to the context of treatment (e.g., among adults already exhibiting cognitive dysfunction) and later-life intervention (e.g., older age). No studies to date have examined the effects of aerobic exercise training in a middle-aged group of at-risk adults, despite recommendations for midlife preventive interventions to target mechanisms of brain health and prevent cerebrovascular and cognitive disease (27).

As such, the purpose of this pilot study was to investigate the effect of 12-wk aerobic exercise training on cerebral pulsatility (primary outcome) and vascular contributors to cerebral pulsatility (large artery stiffness and characteristic impedance, and pulsatile damping) in inactive middle-aged adults with treatment-naïve stage 1 hypertension or elevated blood pressure. This population was chosen because *1*) hypertension is a potent risk factor for later-life cognitive disease (4) and *2*) midlife vascular health is a strong predictor of later-life brain health, making this group a critical population for preventive behavioral lifestyle interventions. We hypothesized that 12 wk of aerobic exercise training would lead to differential changes in cerebral pulsatility compared with control and this would reflect differential changes in arterial stiffness, characteristic impedance, pulse pressure, and pulsatile damping.

## METHODOLOGY

### Participants

Twenty-eight middle-aged adults (40–64 yr of age) with elevated blood pressure (120–129 mmHg systolic) or stage 1 hypertension (130–139 systolic, 80–89 mmHg diastolic) who were not meeting physical activity guidelines (i.e., <150 min/wk of moderate-intensity physical activity and <75 min/wk of vigorous-intensity physical activity; 28) were recruited for this study. Participants did not smoke or vape; had no history of stroke, type I or II diabetes, cardiovascular event, concussion within the last 3 mo, or pulmonary/renal/neurological disease; were not taking antihypertensive medication, hormonal replacement therapy, or oral contraceptives; had a body mass index <40 kg/m^2^; were not currently pregnant; had no significant mobility issues; and did not have dementia (score <21) or mild cognitive impairment (score <26), or depression (defined as PHQ-9 score ≥10). Menopause status was determined via self-report based on the STRAW +10 criteria and guidelines. All participants provided written informed consent prior to study initiation and all procedures were approved by the Iowa State University Institutional Review Board and conformed to the standards outlined in the Declaration of Helsinki.

### Study Design

**Figure 1.**
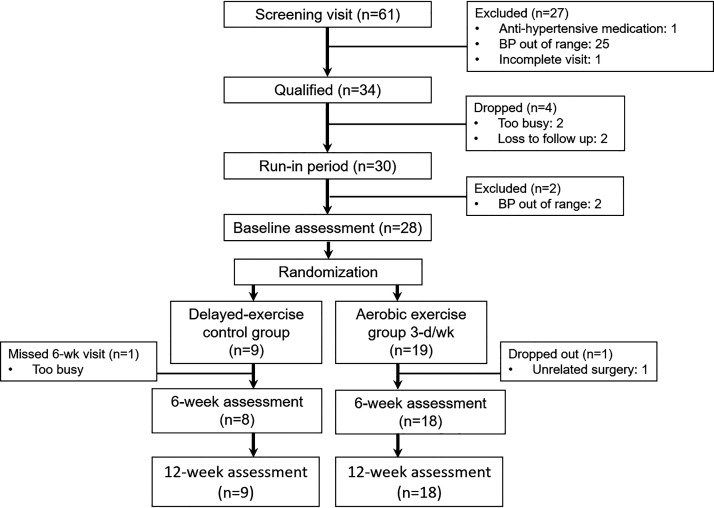
Participant flow chart.

Participants underwent multiple stages of eligibility screening, including an initial online screening, in-person screening, and three run-in visits (Fig. 1). Eligible participants then completed baseline outcome assessments and were randomized to either 12-wk aerobic exercise training (AET) or a waitlist control (CON) in a 2:1 fashion. A 2:1 randomization ratio favoring AET was chosen in order to maximize sample size and obtain a more robust estimation of variation for changes in the primary outcome (MCA PI) with AET to support statistical power analyses on future exercise trials. Outcome assessments were repeated at 6 and 12 wk.

#### Screening process.

Participants completed an online prescreening survey to identify self-reported exclusion criteria, followed by an in-person informed consent and screening to reassess self-reported exclusion criteria (e.g., physical activity levels, health history) and measured exclusion criteria (e.g., body mass index, blood pressure, dementia, depression). Blood pressure was assessed during the in-person screening visit using an automated oscillometric cuff (HEM-907XL OMRON Healthcare, Inc., Lake Forest, IL) following 5 min of quiet, seated rest with feet flat on the floor. Triplicate blood pressures were taken with 1 min of rest between measures while the technician remained outside the room. Values were averaged if two measures were within 5 mmHg or repeated until two measurements achieved this criterion.

#### Run-in visits.

Participants completed three run-in visits within a 7-day period to predict potential adherence to available AET intervention times and continue assessing blood pressure inclusion criteria since a single office blood pressure assessment may be inaccurate to categorize blood pressure levels (29). Run-in visits included familiarization with cerebrovascular measures, cognitive task familiarization and practice (full explanation and practice of each task to mitigate potential variability from learning effects), and lifestyle education on the importance of physical activity. Seated brachial blood pressure was assessed in the same manner as the screening visit (described previously) at all run-in visits. To remain eligible for the baseline visit, participants were required to complete all three run-in visits and have an average blood pressure (averaged across screening and the three run-in visits) within the elevated or stage 1 hypertension range.

#### Randomization and intervention assignment.

Following completion of baseline assessments, participants were randomized based on age, sex, body mass index, and mean arterial pressure at baseline. The technician and principal investigator responsible for cerebrovascular assessments and analyses were blinded to participant assignment.

##### AET intervention.

**Figure 2.**
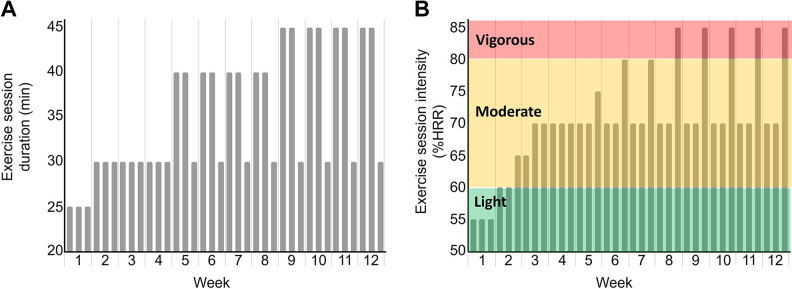
Changes in aerobic exercise session duration (*A*) and intensity during the 12-wk intervention (*B*). HRR, heart rate reserve.

Participants randomized into the AET group attended three supervised exercise sessions per week for 12 wk. Participants could choose to exercise on cycle ergometers, ellipticals, or treadmills and were permitted to switch modalities on a session-by-session basis, but remain on one modality within a single session. Each exercise session included a 5-min warm-up and cool-down. AET progressed to 70%–85% heart rate reserve (HRR) and 30–40 min in duration (Fig. 2). By *week 8* of the AET, participants completed 2 days of 40-min exercise at 70% HRR and 1 day of 30-min exercise at 85% HRR. This AET protocol was based on work by Tomoto et al. (25) that showed a similar prescription could modify cerebral pulsatility over a 12-mo training period but modified exercise frequency and rate of progression to fit our shorter intervention duration. Resting heart rate was reassessed during the 6-wk assessment visit to update HRR calculations.

The aerobic prescription was based on resting HR and maximum HR taken from baseline assessments, including the maximal exercise test on the treadmill. Participants wore a heart rate monitor during each workout that interfaced with their exercise machine (Technogym, Gambettola, Italy) to automatically adjust workload (speed, grade, resistance) and keep the participant in the goal heart rate range for that session. Adherence was measured as the number of sessions attended divided by total number of sessions over the 12-wk intervention. Acceptable adherence to the study protocol was defined as ≥80%.

#### Control intervention.

Participants randomized into the control intervention were asked to maintain their current lifestyle over the 12-wk period and refrain from altering physical activity habits or diet during their enrollment in the study. Participants were given the option to complete the 12-wk AET protocol following the completion of their 12-wk visit to minimize control group dropout.

#### Assessment time points.

Before all visits, participants were required to fast overnight, >12 h abstain from alcohol, exercise, and dietary supplements. In addition, participants abstained from caffeine and nonessential medication (e.g., NSAIDS, nutritional/dietary supplements, allergy medications) the morning of the assessment visits. Outcome measures at baseline and 12 wk included assessment of cerebrovascular and large artery hemodynamics, cognitive function, cardiorespiratory fitness, serum sex hormones, anthropometrics and body composition, physical activity assessment, and a 24-h dietary recall. Outcomes at 6 wk included anthropometrics, body composition, and cerebrovascular measures.

### Outcome Measures

#### Large artery hemodynamics.

##### Blood pressure.

Brachial systolic and diastolic blood pressure were measured in duplicate using an oscillometric cuff and averaged if within 5 mmHg. Carotid and brachial blood pressure waveforms were assessed via tonometry, with waveforms signal averaged across a 20-s epoch. Brachial pressure waveforms were calibrated to brachial systolic and diastolic cuff pressure. Brachial diastolic and mean pressures were derived from brachial pressure waveforms and used to calibrate carotid pressure waveforms to determine carotid systolic, diastolic, and pulse pressure (systolic-diastolic pressure).

#### Large artery stiffness and characteristic impedance.

Aortic and carotid characteristic impedance were calculated in the time domain by dividing the peak derivative of carotid pressure by peak derivative of flow at each site (carotid vs. aorta), as described elsewhere (30). In brief, aortic and common carotid volumetric flow rates were calculated from digitized aortic and common carotid Doppler audio waveforms (31) (described in *Cerebrovascular hemodynamics*) multiplied by the respective artery cross-sectional area. Cross-sectional area was calculated using diastolic vessel diameter (aorta, measured in parasternal long axis view; common carotid, measured longitudinally just proximal to the carotid bifurcation) assuming a circular orifice (30). Proximal aortic compliance was assessed as *A*/[ρ·(Zc·*A*/ρ)^2^], where *A* is aortic cross-sectional area, ρ is a constant 1.06 g/cm^3^ for the density of blood, and Zc is characteristic impedance in the time domain (32).

Large artery stiffness was assessed at the aorta and common carotid arteries. Aortic stiffness was assessed as carotid-femoral pulse wave velocity (cfPWV; NIHem, Cardiovascular Engineering Inc.) via applanation tonometry and simultaneous R-wave gating. CfPWV was calculated as previously described [distance between carotid and femoral sites divided by the time lag (Δ*t*) between carotid-femoral waves] (33). Carotid stiffness was assessed via β-stiffness as ln(systolic pressure/diastolic pressure)/[(systolic diameter – diastolic diameter)/diastolic diameter] using ultrasound (7.5–10.0 MHz linear-array probe; Arietta 70; Hitachi-Aloka) and carotid pressures obtained from contralateral carotid tonometry, described earlier. The timing of carotid diameter and pressure assessments ranged from simultaneously (participants with stronger signals/easier pulse sites) to separately, but within 5 min of each other (participants with more challenging signal acquisition).

#### Cerebrovascular hemodynamics.

Cerebral pulsatility index and mean velocity using transcranial Doppler (TCD) from the left middle (MCA; primary outcome) and right anterior cerebral artery (ACA; secondary outcome, assessed in subset of *n* = 7 control, *n* = 13 AET) using 2-MHz transcranial Doppler probes secured to the temporal window via headset (19) (TOCM Neurovision; Multigon Industries, Inc.) following recommended insonation protocols. The MCA and ACA were used for examining cerebral pulsatility due to their proximity to the common carotid artery. Common carotid pulsatility index and mean velocity were assessed in a similar fashion using pulsed-wave Doppler (Arietta 70; Hitachi-Aloka). MCA and ACA mean velocity and PI were calculated over eight, 7-s epochs across a 2-min period, whereas carotid mean velocity and PI were calculated over two, 12-s epochs. Pulsatility index was calculated as (systolic – diastolic velocity)/mean velocity). Between- and within-day reliability for MCA pulsatility index (our primary outcome) is 3.6% and 3.5%, respectively, within our laboratory. Pulsatile damping was calculated as proximal (i.e., carotid) divided by downstream PI (i.e., MCA, ACA) for MCA and ACA, respectively. MCA and ACA conductance were calculated as mean velocity divided by brachial mean arterial pressure derived from the brachial pressure waveform (described previously). End-tidal CO_2_ was measured during the cerebrovascular assessments via mouthpiece and infrared spectroscopy (AGM100; MediPines Inc.).

#### Cognitive function.

Cognitive function was measured by a 15-min computerized cognitive battery (MATLAB; The MathWorks, Natick, MA; and PsychToolbox) consisting of three tasks (Flanker, 2-Back, memory recognition). Tasks targeted memory, attention (Flanker), and working memory (2-Back) due to their implications for later-life cognitive function. Cognitive testing was completed independently in a quiet room after a verbal and visual reminder of the cognitive battery tasks. Participants were asked to respond as quickly and accurately as possible during the tasks. The cognitive battery began with a word study list and was followed by the attention (Flanker) and working memory (2-back version of an n-back) in a randomized order. The memory recognition task was then completed where individuals were asked to identify words that they recognized from the initial word study list. The task stimuli included the Flanker task, 96 congruent (e.g., ⋘<) and 96 incongruent (e.g., ≪>≪) stimuli; 2-Back, 240 stimuli (24 matches, 216 distractors); memory recognition, 36 studied words and 36 distractors. All stimuli had 1.5-s response windows separated by 0.5-s gaps with presentation of three white crosses (+++) between stimuli. Accuracy was calculated as the number of correct responses divided by the number of total attempted responses. Hit reaction time was averaged across all correct responses within each task. Computer issues/errors during outcome assessment prevented completion of the memory task in two CON and four AET participants; thus, memory task data are presented for *n* = 7 CON and *n* = 14 AET.

#### Cardiorespiratory fitness.

Cardiorespiratory fitness was assessed as peak oxygen uptake (V̇o_2peak_) during an incremental exercise test to volitional fatigue after conclusion of cerebrovascular and cognitive testing. Oxygen consumption was measured via indirect calorimetry using a Moxus metabolic cart (AEI Technologies, Pittsburgh, PA). Participants completed a modified Balke protocol that included a self-selected comfortable walking or jogging pace for a 5-min warm-up at 0% incline, followed by increasing grade by +2.5% every 2 min until 12.5% (after which, speed was increased by +0.2 mph every min). V̇o_2peak_ was assessed as the highest recorded 15-s value when two of the following three criteria were satisfied: *1*) respiratory exchange ratio ≥1.10; *2*) peak heart rate ≥85% of age-predicted max (220-age); or *3*) peak rating of perceived exertion ≥17 on the Borg Scale.

#### Serum sex hormones.

Serum sex hormones were measured at baseline and 12 wk to quantify if there were any mean changes in sex hormones at assessment visits. This was necessary to account for potential changes in sex hormones *1*) from baseline to 12-wk visits since menstrual cycle phase was not standardized among menstruating participants, and *2*) that accompany middle age (e.g., perimenopause) and could vary across a 3-mo period. A venous blood sample was collected via trained phlebotomist and centrifuged at 2,500 rpm for 10 min. Serum testosterone, estradiol, progesterone (Enzo Life Sciences, Inc., Farmingdale, NY), and follicle stimulating hormone (FSH; Eagle Biosciences, Inc., Nashua, NH) were analyzed in duplicate via ELISA according to the manufacturer’s instructions (coefficient of variation of 4.9%, 3.5%, 4.1%, and 2.6% for testosterone, estradiol, progesterone, and FSH kits, respectively).

#### Anthropometrics and body composition.

Waist circumference was assessed at the level of the uppermost lateral border of the illium according to NHANES guidelines and height was measured via stadiometer. Lower-body bioelectrical impedance was used to estimate percent body fat and weight (Tanita sc-331s; Tanita Corp. of America, Inc.).

#### Physical activity monitoring.

Participants were asked to wear wrist-worn actigraphy monitor (wGT3X-BT; Actigraph, Pensacola, FL) for seven consecutive days following the baseline measures and 12-wk visits to account for any changes in unsupervised physical activity behavior. Participants were instructed to wear the device continuously unless interacting with water (e.g., showering, swimming). Participants in the AET intervention removed their activity monitors during their exercise sessions during the first week of the intervention to ensure assessment of physical activity levels outside of the intervention. Steps were calculated using the device’s onboard software. Daily steps and vector magnitude were averaged across the 7 days for analysis.

#### Diet.

Participants completed a 24-h dietary recall (ASA24) the day following their baseline and 12-wk visits. Participants were instructed to recall all food, fluid, and supplement consumption, time of consumption, and amount consumed. Total caloric and fat intake were calculated for analysis.

### Statistical Analyses

All data were analyzed using Statistical Package for the Social Sciences Version 25 (IBM, Inc., Armonk, NY). Normality was assessed quantitatively using the Shapiro–Wilk test, with non-normal data (proximal aortic compliance, and serum testosterone, FSH, progesterone) successfully logarithmically transformed to meet normality assumptions (no nonparametric analyses are necessary; data are still presented as means ± SD from untransformed data but with *P* values and effect sizes derived from log-transformed analyses). Descriptive characteristics between AET and CON were compared using independent *T* tests for continuous variables and χ^2^ tests for categorical variables.

We elected to examine the main effects of 12-wk aerobic exercise training using repeated measures ANOVAs to obtain estimates of effect size to inform future research that are less feasible with linear mixed models. The effect of 12-wk aerobic exercise training on cardiorespiratory fitness, cognitive function, and secondary outcomes was examined via 2 × 2 [2 groups (AET, CON) × 2 times (baseline, 12 wk)] repeated measures ANOVA. The effect of 12-wk aerobic exercise training on cerebrovascular and large artery hemodynamics was examined using 2 × 3 [2 groups (AET, CON) × 3 times (baseline, 6 wk, 12 wk)] repeated measures ANOVA. Significant group-by-time interaction were further explored using Bonferroni corrected post hoc pairwise tests. Flanker task accuracy was non-normally distributed and unable to be successfully transformed to meet normality assumptions. Effect sizes for our main effects are presented as partial eta squared (η^2^) with their corresponding *P* values for ANOVA analyses as is recommended (34). Partial eta squared indicates the percentage of variance in the dependent variable explained by the independent variable or interaction term, with values of 0.01, 0.06, and 0.14 representing small, medium, and large effects, respectively. Data interpretation from this pilot study did not rely solely on *P* values <0.05 (35) and reflects a comprehensive evaluation of *1*) significance level, *2*) effect sizes, and *3*) the totality of evidence for primary and secondary outcomes (34, 36, 37). All data are reported as means ± SD with an a priori α < 0.05.

## RESULTS

Figure 1 displays participant flow from screening to 12-wk testing. Baseline descriptive characteristics are displayed in Table 1. By design, groups were matched for sex, age, and body mass index. No participants reported any medication changes during the intervention. The AET group had high completion (95%, *n* = 1 dropped) and attendance adherence rates (88.4 ± 12.2%, with only 2 of 18 individuals that completed the intervention adhering <80%). No adverse events occurred in either group.

**Table 1.** Descriptive characteristics of the study participants by group assignment

	Total (*n* = 28)	Control (*n* = 9)	AET (*n* = 19)
Age, yr	52 ± 7	51 ± 7	53 ± 7
Sex [female *n* (%)]	19 (68)	6 (67)	13 (68)
Height, cm	170.1 ± 9.7	171.1 ± 7.6	169.6 ± 10.7
Weight, kg	88.2 ± 19.2	89.1 ± 18.5	87.8 ± 20.0
BMI, kg/m^2^	30.2 ± 4.5	30.2 ± 4.6	30.2 ± 4.5
Medication use, *n* (%)			
Statin	1 (3.5)	0 (0.0)	1 (5.3)
Thyroid	3 (10.7)	1 (11.1)	2 (10.5)
Anti-anxiety	9 (32.1)	2 (22.2)	7 (36.8)
SSRI/SNRI/NDRI	7 (17.9)	2 (11.1)	5 (21.1)
Gabapentin	2 (7.1)	1 (11.1)	1 (5.3)
Benzodiazepine	1 (3.6)	0 (0.0)	1 (5.3)
Pregabalin	1 (3.6)	0 (0.0)	1 (5.3)
Tizanidine	1 (3.6)	1 (11.1)	0 (0.0)
Menopause status, *n* (%)			
Premenopausal	8 (42)	2 (33)	6 (46)
Perimenopausal	5 (26)	2 (33)	3 (23)
Postmenopausal	6 (32)	2 (33)	4 (31)
Systolic BP, mmHg^	128 ± 5	130 ± 5	127 ± 6
Diastolic BP, mmHg^	83 ± 5	82 ± 6	83 ± 4
Total cholesterol, mg/dL	204 ± 45	204 ± 21	203 ± 52
HDL, mg/dL	54 ± 13	62 ± 15	51 ± 11
LDL, mg/dL	122 ± 32	123 ± 25	122 ± 35
Glucose, mg/dL	94 ± 8	96 ± 10	93 ± 7

Values are mean ± SD unless otherwise noted. AET *n* = 19, Control *n* = 9. AET, aerobic exercise training group; BMI, body mass index; HDL, high-density lipoprotein; LDL, low-density lipoprotein; NDRI, norepinephrine dopamine reuptake inhibitors; SSRI, selective serotonin reuptake inhibitors, SNRI, serotonin and norepinephrine reuptake inhibitors. ^Averaged brachial blood pressure from screening and run-in visits used to determine eligibility.

Significant group-by-time interactions were observed for relative V̇o_2peak_ (Table 2) which increased at 12 wk (*P* = 0.01) compared with baseline in the AET group. A large effect size was observed for the group-by-time interaction for absolute V̇o_2peak_ (η^2^ = 0.14, *P* = 0.06) which tended to increase in the AET group from baseline to 12 wk, although it did not reach statistical significance criteria. No statistically significant main or interaction effects (and small to medium effect sizes) were observed for weight, body mass index (BMI), or percent body fat. No significant main effects or interactions were detected for changes in serum sex hormones (progesterone, FSH, testosterone, estradiol) between groups during the intervention. Based on dietary recall and physical activity monitoring, diet and physical activity outside of the intervention did not statistically change from baseline to 12 wk with no main or group-by-time interactions for total kilocalories, kilocalories from fat, average steps, or vector magnitude.

**Table 2.** Changes in V̇o_2peak_, anthropometrics, serum sex hormones, 24-h dietary intake, and physical activity in aerobic exercise training vs. control

	Visit	CON	AET	Group	Time	G × T
V̇o_2peak_, L/min	BL	2.6 ± 0.9	2.3 ± 0.6	0.50 (0.02)	0.63 (0.01)	0.06 (0.14)
	12 wk	2.5 ± 0.7	2.5 ± 0.6			
V̇o_2peak_, mL/kg/min	BL	29.4 ± 7.5	27.0 ± 5.4	0.68 (0.01)	0.54 (0.02)	0.01 (0.22)
	12 wk	28.4 ± 8.0	28.6 ± 6.0*			
Weight, kg	BL	89.1 ± 18.5	88.0 ± 20.5	0.86 (0.001)	0.28 (0.05)	0.30 (0.04)
	12 wk	89.1 ± 17.2	87.4 ± 20.8			
BMI, kg/m^2^	BL	30.2 ± 4.5	30.1 ± 4.6	0.91 (0.001)	0.54 (0.01)	0.26 (0.05)
	12 wk	30.3 ± 4.3	29.9 ± 4.8			
Body fat, %	BL	37.7 ± 11.1	38.1 ± 8.6	1.00 (0.001)	0.57 (0.01)	0.30 (0.04)
	12 wk	38.3 ± 10.5	38.0 ± 9.0			
Progesterone, ng/mL^	BL	1.87 ± 0.41	2.18 ± 1.46	0.75 (0.01)	0.92 (0.001)	0.66 (0.01)
	12 wk	2.07 ± 0.82	2.14 ± 1.43			
FSH, IU/mL^	BL	23.0 ± 22.4	30.8 ± 40.3	0.76 (0.001)	0.76 (0.001)	0.12 (0.10)
	12 wk	21.6 ± 23.7	36.5 ± 40.5			
Testosterone, pg/mL^	BL	272.3 ± 383.1	293.8 ± 408.3	0.98 (0.001)	0.43 (0.03)	0.23 (0.06)
	12 wk	230.9 ± 322.3	284.6 ± 409.3			
Estradiol, pg/mL^	BL	32.8 ± 13.6	36.3 ± 15.3	0.56 (0.01)	0.54 (0.02)	0.90 (0.001)
	12 wk	30.9 ± 10.5	35.0 ± 18.7			
Total caloric intake, kcal	BL	1,879 ± 688	2,049 ± 805	0.47 (0.02)	0.72 (0.005)	0.97 (0.001)
	12 wk	1,933 ± 786	2,117 ± 637			
Total fat intake, kcal	BL	87 ± 26	95 ± 42	0.24 (0.06)	0.83 (0.002)	0.62 (0.01)
	12 wk	80 ± 33	97 ± 35			
7-day mean steps (×10^3^)	BL	9.4 ± 2.2	9.8 ± 2.7	0.86 (0.001)	0.34 (0.04)	0.64 (0.01)
	12 wk	10.2 ± 2.7	10.1 ± 2.0			
7-day vector magnitude (×10^5^)	BL	21.2 ± 4.6	21.6 ± 6.9	0.86 (0.001)	0.73 (0.005)	0.51 (0.02)
	12 wk	22.4 ± 5.8	21.2 ± 4.8			

Values are mean ± SD, with main effects reported as *P* (partial η^2^). AET *n* = 18; Control *n* = 9. AET, aerobic exercise training group; BL, baseline; BMI, body mass index; FSH, follicle stimulating hormone; G × T, group-by-time interaction. ^*n* = 7 control, 18 AET. *P < 0.05 vs. within-condition baseline. Statistical effects tested via repeated-measures ANOVA.

### Blood Pressure

Significant group-by-time interactions were observed for brachial diastolic and mean arterial blood pressure (Table 3). Diastolic blood pressure decreased at 6 and 12 wk (*P* = 0.01) compared with baseline in the AET group, whereas post hoc pairwise comparisons for changes in mean arterial pressure were not statistically significant. A large effect size was observed for the group-by-time interaction for heart rate (*P* = 0.06, η^2^ = 0.11) wherein heart rate tended to decrease in the AET group from baseline to 12 wk, although these did not reach statistical significance of *P* < 0.05. No statistically significant main or interaction effects (and small to medium effect sizes) were observed for brachial systolic blood pressure and ETCO_2_. Changes in carotid systolic pressure (*P* = 0.10, η^2^ = 0.09) and pulse pressure (*P* = 0.10, η^2^ = 0.10) were not statistically significant but showed medium-large effect size increases from baseline and 6–12 wk in both groups.

**Table 3.** Changes in blood pressure, heart rate, and end-tidal CO_2_ in aerobic exercise training vs. control

	Visit	CON	AET	Group	Time	G × T
Systolic blood pressure, mmHg	BL	124 ± 10	127 ± 13	0.65 (0.01)	0.24 (0.06)	0.43 (0.03)
	6 wk	122 ± 9	125 ± 14			
	12 wk	126 ± 8	126 ± 11			
Diastolic blood pressure, mmHg	BL	76 ± 9	82 ± 9	0.43 (0.03)	0.45 (0.03)	0.01 (0.17)
	6 wk	77 ± 6	78 ± 8*			
	12 wk	79 ± 8	78 ± 8*			
Mean blood pressure, mmHg	BL	95 ± 10	102 ± 10	0.43 (0.03)	0.31 (0.05)	0.03 (0.14)
	6 wk	96 ± 7	99 ± 9			
	12 wk	99 ± 8	99 ± 9			
Heart rate, beats/min	BL	61 ± 8	67 ± 9	0.49 (0.02)	0.09 (0.10)	0.06 (0.11)
	6 wk	63 ± 10	64 ± 8			
	12 wk	61 ± 7	62 ± 8			
End-tidal CO_2_, mmHg	BL	41 ± 2	41 ± 5	0.76 (0.004)	0.77 (0.01)	0.80 (0.01)
	6 wk	41 ± 2	40 ± 3			
	12 wk	41 ± 3	41 ± 4			
Carotid systolic pressure, mmHg	BL	121 ± 12	126 ± 14	0.60 (0.01)	0.10 (0.09)	0.34 (0.04)
	6 wk	121 ± 8	124 ± 15			
	12 wk	126 ± 11	126 ± 13			
Carotid pulse pressure, mmHg	BL	44 ± 7	43 ± 10	0.99 (0.001)	0.10 (0.10)	0.62 (0.02)
	6 wk	43 ± 6	44 ± 13			
	12 wk	47 ± 9	47 ± 11			

Values are mean ± SD, with main effects reported as *P* (partial η^2^). AET *n* = 18; Control *n* = 8. AET, aerobic exercise training group; BL, baseline; G × T, group-by-time interaction. *P < 0.05 vs. within-condition baseline. Statistical effects tested via repeated-measures ANOVA.

### Central Vascular Hemodynamics

**Figure 3.**
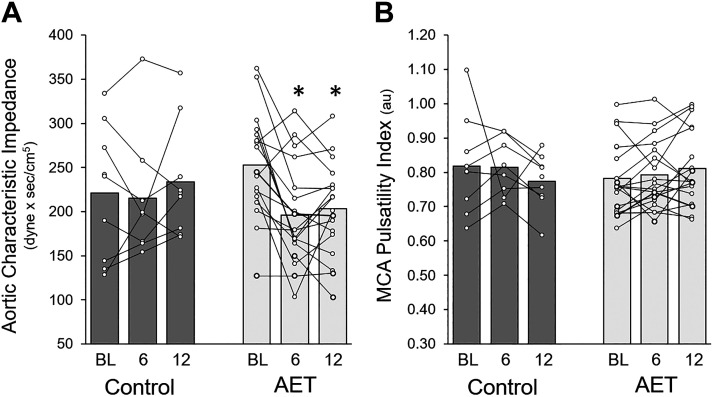
Changes in middle cerebral artery pulsatility index (*A*) and aortic characteristic impedance in control vs. AET at baseline, 6, and 12 wk (*B*). *A*: MCA PI [*n* = 8 control, 18 AET: group 0.82 (0.002), time 0.85 (0.01), group × time 0.07 (0.11)]. *B*: aortic characteristic impedance [*n* = 8 control, 17 AET: group 0.91 (0.001), time 0.04 (0.14), group × time 0.02 (0.17)]. AET, aerobic exercise training; BL, baseline; MCA, middle cerebral artery. *P < 0.05 vs. BL. Main effects, *P* (partial η^2^). Statistical effects tested via repeated-measures ANOVA.

Significant group-by-time interactions were identified for aortic characteristic impedance (*P* = 0.02, η^2^ = 0.17; Fig. 3*A*) and proximal compliance (*P* = 0.04, η^2^ = 0.14), where aortic characteristic impedance decreased, and proximal aortic compliance increased, at 6 and 12 wk compared with baseline in the AET group (Table 4). Large, although not statistically significant, group-by-time interaction effect sizes were observed for cfPWV (*P* = 0.09, η^2^ = 0.10), with cfPWV tending to increase in the CON group from baseline to 12 wk but not in the AET group. A significant time effect was observed for carotid characteristic impedance (*P* = 0.04, η^2^ = 0.13) that increased from baseline to 12 wk in both groups. Effect sizes for carotid β-stiffness, cfPWV relative to MAP, and aortic or common carotid artery diameter were small to small-medium (η^2^ = 0.01–0.05) and not statistically significant.

**Table 4.** Changes in aortic and carotid artery hemodynamics in aerobic exercise training vs. control

	Visit	CON	AET	Group	Time	G × T
Aorta					
Diameter, cm^	BL	1.86 ± 0.09	1.83 ± 0.20	0.87 (0.001)	0.75 (0.01)	0.47 (0.03)
	6 wk	1.84 ± 0.19	1.85 ± 0.19			
	12 wk	1.88 ± 0.20	1.86 ± 0.20			
Characteristic impedance, dyn·s/cm^5^^	BL	219 ± 81	253 ± 60	0.91 (0.001)	0.04 (0.14)	0.02 (0.17)
	6 wk	215 ± 72	199 ± 58*			
	12 wk	235 ± 68	209 ± 48*			
Proximal compliance, 10^−6^ cm^4^/dyn^	BL	7.1 ± 3.4	5.3 ± 1.8	0.55 (0.02)	0.28 (0.05)	0.04 (0.14)
	6 wk	6.9 ± 2.5	6.9 ± 2.6*			
	12 wk	6.3 ± 2.3	6.4 ± 2.3*			
cfPWV, cm/s	BL	728.6 ± 66.1	826.7 ± 166.9	0.40 (0.03)	0.15 (0.08)	0.09 (0.10)
	6 wk	759.0 ± 201.9	807.2 ± 136.2			
	12 wk	811.2 ± 172.2	817.7 ± 133.4			
cfPWV relative to MAP, cm/s/mmHg	BL	7.74 ± 1.15	8.13 ± 1.37	0.64 (0.01)	0.46 (0.03)	0.74 (0.01)
	6 wk	7.91 ± 2.08	8.18 ± 1.03			
	12 wk	8.16 ± 1.53	8.23 ± 0.98			
Carotid						
Diameter, cm^	BL	0.56 ± 0.05	0.57 ± 0.05	0.63 (0.01)	0.27 (0.06)	0.72 (0.01)
	6 wk	0.55 ± 0.06	0.56 ± 0.06			
	12 wk	0.56 ± 0.05	0.56 ± 0.05			
Characteristic impedance, dyn·s/cm^5^^	BL	4,084 ± 1,244	4,930 ± 1,860	0.44 (0.03)	0.04 (0.13)^a^	0.60 (0.02)
	6 wk	4,646 ± 1,777	5,143 ± 1,853			
	12 wk	5,070 ± 1,383	5,369 ± 1,950			
β-Stiffness, au	BL	7.73 ± 2.79	7.15 ± 1.26	0.68 (0.01)	0.30 (0.05)	0.31 (0.05)
	6 wk	6.24 ± 1.78	7.14 ± 1.74			
	12 wk	6.91 ± 1.70	7.25 ± 1.83			
Pulsatility index, au	BL	1.50 ± 0.19	1.32 ± 0.22	0.33 (0.40)	0.60 (0.02)	0.06 (0.11)
	6 wk	1.42 ± 0.28	1.34 ± 0.24			
	12 wk	1.42 ± 0.19	1.41 ± 0.25			

Values are mean ± SD, with main effects reported as *P* (partial η^2^). AET *n* = 18; Control *n* = 8. AET, aerobic exercise training group; BL, baseline; cfPWV, carotid-femoral pulse wave velocity; G × T, group-by-time interaction; MAP, mean arterial pressure. ^a^Time effect *P* < 0.05 for 12 wk vs. BL. ^*n* = 8 control, 17 AET. *P < 0.05 vs. within-condition baseline. Statistical effects tested via repeated-measures ANOVA.

### Cerebrovascular Hemodynamics

Large but not statistically significant effect sizes were observed for group-by-time interactions for MCA PI (η^2^ = 0.11, *P* = 0.07; Fig. 3*B*, Table 5), and CCA PI (η^2^ = 0.11, *P* = 0.06; Table 4) with effects driven by divergent increases in MCA PI and carotid PI from baseline to 12 wk in the AET group and decreases among the CON group. A similar large but not statistically significant group-by-time effect was noted for MCA conductance (η^2^ = 0.11, *P* = 0.06; Table 5), which was driven by reductions in conductance from baseline to 12 wk among CON, coupled with a slight increase in conductance among AET. Main effects for ACA PI and conductance, and MCA and ACA damping all had small effect sizes that were not statistically significant (ACA hemodynamics presented in Supplemental Table S1).

**Table 5.** Changes in middle cerebral artery hemodynamics in aerobic exercise training vs. control

	Visit	CON	AET	Group	Time	G × T
Mean velocity, cm/s	BL	52 ± 10	50 ± 13	0.60 (0.01)	0.93 (0.003)	0.21 (0.06)
	6 wk	53 ± 12	48 ± 11			
	12 wk	50 ± 10	50 ± 11			
Systolic velocity, cm/s	BL	80 ± 15	75 ± 19	0.54 (0.02)	0.82 (0.01)	0.06 (0.11)
	6 wk	82 ± 20	73 ± 18			
	12 wk	76 ± 15	77 ± 17			
Diastolic velocity, cm/s	BL	38 ± 8	37 ± 10	0.59 (0.01)	0.96 (0.002)	0.39 (0.04)
	6 wk	39 ± 6	35 ± 8			
	12 wk	37 ± 7	36 ± 8			
Pulsatility index, au	BL	0.82 ± 0.15	0.78 ± 0.10	0.82 (0.002)	0.85 (0.01)	0.07 (0.11)
	6 wk	0.81 ± 0.08	0.79 ± 0.10			
	12 wk	0.77 ± 0.08	0.81 ± 0.11			
Conductance, cm/s/mmHg	BL	0.55 ± 0.12	0.49 ± 0.11	0.31 (0.04)	0.70 (0.02)	0.06 (0.11)
	6 wk	0.55 ± 0.13	0.49 ± 0.09			
	12 wk	0.51 ± 0.13	0.51 ± 0.11			
Pulsatile damping, au	BL	1.88 ± 0.44	1.73 ± 0.38	0.54 (0.02)	0.39 (0.04)	0.60 (0.02)
	6 wk	1.78 ± 0.47	1.73 ± 0.38			
	12 wk	1.87 ± 0.38	1.77 ± 0.44			

Values are mean ± SD, with main effects reported as *P* (partial η^2^). AET *n* = 18; Control *n* = 8. AET, aerobic exercise training group; BL, baseline; cfPWV, carotid-femoral pulse wave velocity; G × T, group-by-time interaction; MAP, mean arterial pressure. ^*n* = 8 control, 17 AET. ^a^Time effect *P* < 0.05 for 12 wk vs. BL. *P < 0.05 vs. within-condition baseline. Statistical effects tested via repeated-measures ANOVA.

### Cognitive Function

A significant group-by-time interaction was observed for n-back hit reaction time (*P* = 0.02, η^2^ = 0.20), with reaction time decreasing from baseline to 12 wk in the AET group only (Table 6). Main and interaction effects for Flanker and memory task accuracy and reaction times had small effect sizes (η^2^ = 0.00–0.04) and were not statistically significant.

**Table 6.** Changes in cognitive task performance in aerobic exercise training vs. control

	Visit	CON	AET	G	T	G × T
Flanker incongruent hits, %	BL	96 ± 5	97 ± 3	0.78 (0.003)	0.83 (0.002)	0.77 (0.003)
	12 wk	96 ± 3	96 ± 3			
Flanker incongruent reaction time, ms	BL	559 ± 75	591 ± 99	0.37 (0.03)	0.82 (0.002)	0.78 (0.003)
	12 wk	555 ± 73	591 ± 103			
Memory hits, %^	BL	56 ± 18	50 ± 22	0.42 (0.03)	0.74 (0.006)	0.85 (0.02)
	12 wk	58 ± 14	51 ± 19			
Memory false alarms, %^	BL	26 ± 13	29 ± 28	0.81 (0.003)	0.92 (0.001)	0.82 (0.003)
	12 wk	28 ± 20	28 ± 15			
Memory reaction time, ms^	BL	776 ± 124	842 ± 189	0.04 (0.40)	0.24 (0.07)	0.99 (0.00)
	12 wk	805 ± 105	870 ± 165			
2-Back hits, %	BL	64 ± 15	61 ± 18	0.35 (0.04)	0.32 (0.04)	0.54 (0.02)
	12 wk	71 ± 19	63 ± 15			
2-Back reaction time, ms	BL	585 ± 104	640 ± 91	0.60 (0.01)	0.86 (0.001)	0.02 (0.20)
	12 wk	620 ± 78	601 ± 83*			

Values are mean ± SD, with main effects reported as *P* (partial η^2^). AET *n* = 18; Control *n* = 9. AET, aerobic exercise training group; BL, baseline; G × T, group-by-time interaction. ^*n* = 7 control, 14 AET. *P < 0.05 vs. within-condition baseline. Statistical effects tested via repeated-measures ANOVA.

## DISCUSSION

Novel elements of the current study included utilization of 12-wk aerobic exercise training as a midlife preventive strategy to target emerging vascular targets of brain health (cerebral pulsatility and its hemodynamic mechanisms) in middle-aged adults with risk factors for later-life cognitive dysfunction, an understudied, but critical population to attenuate later-life cognitive and cerebrovascular disease burden. Our main findings were that compared with the CON group, AET elicited *1*) reductions in aortic characteristic impedance, increases in proximal aortic compliance, and maintained aortic stiffness; *2*) large paradoxical effects on cerebral pulsatility with increases in middle cerebral artery and common carotid artery pulsatility and no change in cerebral pulsatile damping; and *3*) select improvements in cognitive function with faster working memory reaction times. Cumulatively, these data suggest the potential for differing adaptation rates in the central versus cerebrovasculature that may modestly increase cerebral pulsatility without disrupting cognitive benefits following 12-wk aerobic exercise training.

We noted that 12-wk aerobic exercise training elicited generally beneficial changes in central vascular hemodynamics compared with the control condition. Although 12-wk aerobic exercise training prevented increases in cfPWV seen in the CON group, cfPWV was statistically unchanged compared with baseline in the AET group. This observation aligns with the literature which suggests that aerobic exercise training-induced reductions in cfPWV are highly dependent on substantial reductions in blood pressure (which were modest in the current study, ∼3 mmHg for mean pressure) (38, 39). Despite a maintenance of cfPWV with aerobic exercise training, we noted significant increases in proximal aortic compliance which is in line with previous cross-sectional observations of increased aortic distensibility/compliance in individuals with greater aerobic exercise training history (40, 41). Increases in proximal aortic compliance would be expected to support reductions in aortic characteristic impedance, which we observed in the AET group only. Limited available data (2 single-arm exercise trials, *n* ≤ 10) suggest characteristic impedance may increase in young adults (42), but remain unchanged in older adults with isolated systolic hypertension (43) following 8 wk of aerobic exercise training. Taken together, current data suggest the beneficial effect of aerobic exercise training on central vascular hemodynamics may be age dependent, with middle age being associated with more central vascular (as opposed to cardiac) adaptations to training (44) but the benefits of aerobic exercise on proximal aortic stiffness diminishing with age (41).

Inherent biomechanical differences in the structural properties throughout the length of the aorta (45, 46) may alter how different segments (proximal aorta vs. longer segments assessed via cfPWV) respond to aerobic exercise training. Aortic stiffness, assessed as cfPWV, may be more resistant to change than other regional measures of stiffness since cfPWV includes portions of the peripheral vasculature that may not respond to aerobic exercise training (47). Although most data focus on cfPWV, aortic characteristic impedance may be a more sensitive metric of central hemodynamics to changes with aerobic exercise training. Unlike cfPWV, characteristic impedance is derived from relations between pressure, diameter, and blood velocity, all of which may change with exercise training (48). Even small, nonstatistically significant increases in aortic diameter with aerobic exercise training [as observed herein, and elsewhere (48)] could contribute to changes in characteristic impedance when combined with modest changes in blood pressure, blood velocity, and proximal aortic compliance. Ultimately, increases in aortic compliance and reductions in characteristic impedance from 12-wk aerobic exercise training would be expected to beneficially alter pulsatile hemodynamics.

We noted effect sizes, although not statistically significant, for increases in both carotid and middle cerebral artery pulsatility following 12-wk aerobic exercise training. These findings concur with the very limited, recent data in this area (12-wk moderate-intensity aerobic exercise training in young healthy adults) (49). Contrastingly, aerobic exercise-based interventions 4–6 mo in duration in various populations [young collegiate athletes (50), cognitively healthy middle-aged/older adults (51), and older adults with cerebral small vessel disease (52)], all indicate no changes in cerebral pulsatility. Finally, 12-mo aerobic exercise interventions show mixed results, with no changes in healthy older adults (24) but decreased (e.g., improved) cerebral pulsatility in older adults with mild cognitive impairment (25). Cumulatively, these data indicate the effect of aerobic exercise on cerebral pulsatility may be dependent on the population (age, health status, etc.) and intervention duration. Shorter duration interventions (e.g., ∼12 wk) appear to transiently increase cerebral pulsatility (49), which subsides by 4–6 mo (50–52) and may decrease after 12 mo of training (25). It is possible that exercise intervention duration and study population impact the rate and degree to which vascular contributors to cerebral pulsatility adapt to aerobic exercise training.

The mechanisms underlying the increases in cerebral pulsatility observed herein are unclear but may stem in part from faster rates of adaptation to aerobic exercise in the central versus cerebrovasculature. Although stroke volume was not directly measured in this study, we noted large effects for reductions in heart rate in the AET group [potentially indicative of hallmark increases in stroke volume following training (53–55)]. Improvements in proximal aortic compliance and characteristic impedance may have allowed the aorta to absorb a larger stroke volume without drastically increasing aortic pulsatility, but this was not apparent for the cerebrovasculature (no AET-specific change in carotid stiffness/characteristic impedance or MCA pulsatile damping). In this scenario, enhanced contractility and the ejection of a larger stroke volume into a carotid artery and cerebrovasculature not yet adapted to accommodate it could increase the transmission of pulsatility (greater forward wave energy, greater pulsatile power) into the cerebrovasculature (56) and increase MCA systolic blood velocity and pulsatility. Alternatively, modest reductions in mean pressure and concomitant cerebral autoregulatory changes may alter the mechanoelastic properties of the large cerebral arteries and augment cerebral pulsatility (57) via reductions in diastolic blood velocity. Longer duration exercise interventions may maximize cerebrovascular adaptations that serve to limit cerebral pulsatility [cerebrovascular adaptation to a new regulatory set point at a lower blood pressure, carotid destiffening (24), and enhanced cerebral pulsatile damping (observed cross-sectionally; (22)]; however, this requires additional investigation.

## IMPLICATIONS

Our data suggest 12 wk of aerobic exercise training may modestly increase cerebral pulsatility. In the context of aging, greater cerebral pulsatility over years of exposure is hypothesized to damage the brain and contribute to cerebrovascular and cognitive disease (17, 20, 21). If the modest increases in cerebral pulsatility within our 12-wk exercise intervention had similar negative effects as those seen with aging, we might expect to see impairments in cognitive function following our intervention. Instead, we noted beneficial acceleration of working memory reaction time, suggesting that the modest increase in cerebral pulsatility following 12-wk aerobic exercise training did not prevent beneficial improvements in select metrics of cognitive function. As such, we posit that the initial, modest increase in cerebral pulsatility with shorter duration aerobic exercise training is not detrimental, but rather a reflection of different adaptation rates between segments of the vascular tree (central vs. cerebral) since cerebral pulsatility has not been found to increase in longer duration interventions (50–52). Future studies are required to better understand potential temporal differences in adaptation rates to aerobic exercise between the heart, central vasculature, and cerebrovasculature.

## LIMITATIONS

We wish to underscore that this was a pilot study with a short-term 3-mo aerobic exercise training intervention that used a 2:1 randomization favoring exercise to identify and quantify the magnitude of the effects (effect size and variation) of aerobic exercise training on cerebral pulsatility to inform future research. Owing to the limited available literature and the nature of this pilot study, no formal a priori power calculation was conducted to estimate the sample size of this study, although our AET sample size was comparable to the lone randomized exercise trial at the time that had examined cerebral pulsatility (25). Longer duration interventions (e.g., 6–12 mo) may be necessary to better understand differing temporal adaptations among vascular contributors to cerebral pulsatility. The large amount of data presented herein as part of our exercise trial, combined with our modest sample size, may increase the chance of type I and II errors. It should be underscored, however, that all data presented herein were key for a comprehensive approach to assess cerebral pulsatility and its vascular contributors and assess potential exercise trial confounders (e.g., sex hormones, physical activity, diet).

Due to the nature of this study longitudinal exercise intervention, we were not able to standardize outcome assessments for menstrual cycle in our female participants at our three assessment visits since standardizing visits to early follicular would potentially extend the exercise intervention duration (and thus adaptation stimulus) for individuals with irregular (e.g., perimenopausal) or >30-day cycles. Statistically covarying for changes in sex hormones did not alter the magnitude of effects on cerebral pulsatility, suggesting that potential changes in sex hormones from unstandardized visits among menstruating females did not substantially alter our findings (analyses not shown). In addition, using linear mixed models and the intent-to-treat principle, and excluding the *n* = 2 individuals with <80% adherence to the AET program, did not substantially alter our findings and the interpretation of our data (data not shown). Further work is necessary to understand if changes in cerebral pulsatility differ by intracranial vessel (e.g., MCA vs. ACA). We generally noted attenuated effects of aerobic exercise training on ACA pulsatility (see Supplemental Table S1) compared with the MCA and it is unclear if it is a true disparate response or if it reflects inadequate power owing to the subsample that completed ACA hemodynamic assessment (*n* = 7 control, *n* = 13 AET). Aortic characteristic was measured using carotid, rather than aortic pressure waveforms, which may result in a small 1%–3% overestimation of aortic characteristic impedance. As such, our absolute aortic characteristic impedance values may be slightly higher than other studies using measured aortic pressure waveforms. Future studies need to further investigate brain structural and functional data (e.g., via MRI) to close the mechanistic gap in knowledge between the effects of aerobic exercise on cerebral hemodynamics and cognitive performance.

### Conclusions

Our data suggest that 12 wk of AET lead to beneficial changes in the central vasculature (increased proximal aortic compliance, decreased aortic characteristic impedance) but paradoxical, modest increases in cerebral pulsatility in middle-aged adults with stage 1 or elevated blood pressure. Ultimately, the modest increase in cerebral pulsatility elicited following 12-wk aerobic exercise training herein does not appear to be detrimental as it did not prevent beneficial changes in cognitive function (accelerated working memory reaction time). Further research is necessary to understand whether different adaptation rates in the central versus cerebral vasculature contribute to changes in cerebral pulsatility with aerobic exercise training and how this differs in healthy, at-risk, and clinical populations.

## DATA AVAILABILITY

Data will be made available upon reasonable request.

## SUPPLEMENTAL DATA

10.6084/m9.figshare.24168687 Supplemental Table S1: https://doi.org/10.6084/m9.figshare.24168687.

## GRANTS

The data presented in this manuscript were funded by an Iowa State University College of Human Sciences (Faculty Seed Grant, to W.K.L.).

## DISCLOSURES

No conflicts of interest, financial or otherwise, are declared by the authors.

## AUTHOR CONTRIBUTIONS

W.K.L. conceived and designed research; A.M.F., Q.K., and W.K.L. performed experiments; K.S.R., A.M.F., Q.K., and W.K.L. analyzed data; K.S.R., A.G.B., and W.K.L. interpreted results of experiments; K.S.R., A.M.F., and W.K.L. prepared figures; K.S.R., A.M.F., and W.K.L. drafted manuscript; K.S.R., A.M.F., Q.K., A.G.B., M.L.K., and W.K.L. edited and revised manuscript; K.S.R., A.M.F., Q.K., A.G.B., M.L.K., and W.K.L. approved final version of manuscript.
